# Risk of complications in post‐term pregnancies: Spontaneous labor should not be included in the intervention group

**DOI:** 10.1111/aogs.14330

**Published:** 2022-02-24

**Authors:** Ole Bredahl Rasmussen, Finn Friis Lauszus

**Affiliations:** ^1^ Department of Obstetrics and Gynecology Herning Hospital Herning Denmark; ^2^ Department of Obstetrics and Gynecology Aabenraa Hospital Aabenraa Denmark

## Abstract

Spontaneous labor should not be included in the intervention group when discussing induction of labor vs expectant management in post‐term pregnancies.

Sir,

Congratulations on an exciting and timely report concerning induction of labor (IOL) in post‐term pregnancies.^1^ In your article, you conclude that “births at GA 41+4 to 42+0 (“late group”) were associated with an increased risk of neonatal morbidity and birth complications compared with births at GA 41+0 to 41+3 weeks (“early group”) (our quotation marks). The intent of your analysis was to “determine the independent impact of GA” and thus “aid clinicians in deciding when to recommend IOL in late‐term pregnancies”.

It is well known that the rate of cesarean section (CS) in spontaneous labor is lower compared with the CS rate in IOL in the same gestational week (GA),^2^ but this type of comparison is not relevant to the clinician and the pregnant woman when choosing between IOL or expectant management.^3^ Likewise, it is fundamental that the intervention group only includes women with IOL in a comparison between IOL and expectant management.

In your case, the intervention group or the early group is comprised of both women with IOL and women with a spontaneous labor. This means that the data from the early period will be influenced by the impact of the group of women with spontaneous labor. The regression analysis may adjust for but not exclude the influence on the outcome by the group of women with spontaneous labor. Thus, your method will inevitably bias against expectant management.

Previously, one way was recommended by Caughey et al to carry out an analysis of retrospective data with focus on the clinical aspect of IOL.^4^ They compared IOL at the current gestational week vs expectant management with birth at a greater gestational week. Furthermore, using this method on Danish data, no added risk of CS was found in IOL from GA 39 to 41 compared with expectant management.^5^ Analyzed in this way, your data may give valuable information on the influence of IOL vs expectant management in post‐term pregnancies, which is the clinical question.
